# Petal Cellular Identities

**DOI:** 10.3389/fpls.2021.745507

**Published:** 2021-10-27

**Authors:** Quentin Cavallini-Speisser, Patrice Morel, Marie Monniaux

**Affiliations:** Laboratoire de Reproduction et Développement des Plantes, Université de Lyon, ENS de Lyon, UCB Lyon 1, CNRS, INRAE, Lyon, France

**Keywords:** petal, cell type, conical cell, mesophyll, epidermis, cell identity, petal polarities

## Abstract

Petals are typified by their conical epidermal cells that play a predominant role for the attraction and interaction with pollinators. However, cell identities in the petal can be very diverse, with different cell types in subdomains of the petal, in different cell layers, and depending on their adaxial-abaxial or proximo-distal position in the petal. In this mini-review, we give an overview of the main cell types that can be found in the petal and describe some of their functions. We review what is known about the genetic basis for the establishment of these cellular identities and their possible relation with petal identity and polarity specifiers expressed earlier during petal development, in an attempt to bridge the gap between organ identity and cell identity in the petal.

## Introduction

Diversity in petal shape, size, color, and number is a key contributor to the dazzling variety of floral forms observed in the wild. The petal is often described as a very simple laminar structure, reminiscent of a leaf in its shape. The *Arabidopsis* petal could not be much simpler: a flat organ with a basal greenish claw and a distal white blade and only few different cell types (Irish, 2008). This simplicity makes it an excellent model to study plant organogenesis and cell type differentiation processes (Irish, 2008; Szécsi et al., 2014; Huang and Irish, 2016). However, *Arabidopsis* is only one among more than 350,000 flowering plant species (The Plant List, 2013), whose petal structures can be much more complex (Endress, 2001; Moyroud and Glover, 2017). Petals can display complex elaborations, such as lobes, fringes, nectary spurs, or hair pads (Endress and Matthews, 2006). In most asterid species, petals are fused together; therefore, the proximal (tube) and distal (limbs) parts of the fused petals can appear very different (Endress, 2001). Moreover, within a single flower, all petals are not the same, particularly in bilaterally symmetric flowers: Legume flowers develop distinct dorsal, lateral, and ventral petals (Ojeda et al., 2009). Petals also display an abaxial-adaxial polarity, the adaxial side of the petal being the upper/inner one (closest to the main stem), while the abaxial side is the lower/outer one. Finally, petal cells also have a layer identity, since petals generally derive from 2 (sometimes 3) layers from the shoot apical meristem that generates all aerial organs (Satina and Blakeslee, 1941; Jenik and Irish, 2000). Mature petals are thus typically composed of an adaxial epidermal layer (L1-derived), a few layers of mesophyll cells (L2-derived), and an abaxial epidermal layer (L1-derived).

In this mini-review, we will give an overview of the diversity of cell types that can be encountered on this apparently simple structure that is the petal. We will first focus on the two petal epidermises in which we find conical cells, together with many other cell types. We will next explore cell types and functions in the petal mesophyll, containing the petal vasculature surrounded by parenchyma cells. Finally, we will review the molecular mechanisms involved in cell differentiation in the petal epidermis and their potential link with petal identity and polarity specifiers.

## The Petal Epidermis: Conical Cells, Striations, Trichomes, and Stomata

Petal epidermal cells display striking differentiation features. The typical petal epidermal cell is conical (also called papillate), and this particular cell shape, readily observable by light microscopy or scanning electron microscopy, is often used as a marker for petal cell identity; indeed, it is found in 75–80% of angiosperm petals (Kay et al., 1981). Conical cells are generally found on the adaxial (upper) surface of the petal limb, and their shape and size can be extremely different among angiosperm species (Kay et al., 1981; Whitney et al., 2011a). They have been shown to increase petal color intensity and cause its sparkling appearance, increase pollinator’s grip on the flower, affect overall petal shape, and decrease its wettability (Gorton and Vogelmann, 1996; Baumann et al., 2007; Whitney et al., 2009a, 2011a,b). They are also in most cases where pigments are produced (Kay et al., 1981) and frequently where scent is released (Baudino et al., 2007). All of the aforementioned traits potentially improve attraction and interaction with pollinators and therefore likely lead to a higher pollination success (Whitney et al., 2011a). Conical cells can thus be viewed as a key cellular innovation of flowering plants.

**Figure 1:**
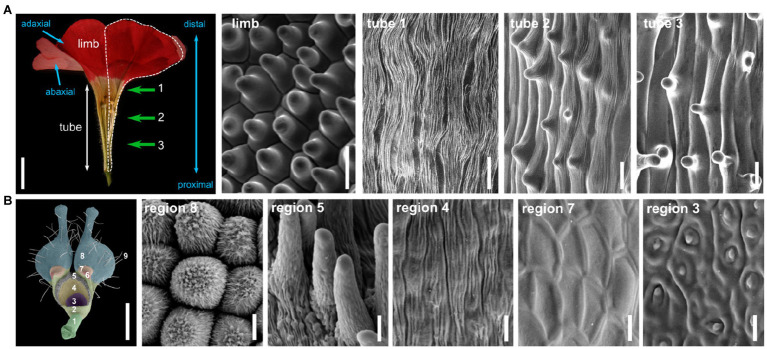
Cellular identities in the petal epidermis. **(A)** Half-flower from *Petunia x hybrida*, cut open longitudinally to display the tube and limb regions (scale bar=1cm). The contour of one petal is shown with a white dotted line. Proximo/distal and adaxial/abaxial polarities of the petal are indicated with blue arrows. Scanning electron micrographs (scale bars=20μm) of the adaxial surface of petals in the limbs, and at three different regions from the tube, indicated by green arrows and numbers in the flower picture. These pictures were obtained with a HIROX SH-1500 bench top environmental scanning electron microscope equipped with a cooling stage (−10°C, 5kV). **(B)** Petal from *Nigella arvensis* viewed from its adaxial side (scale bar=1mm), with nine regions with different cellular identities as identified in Yao et al. (2019). A 10th region is only visible on the abaxial side of the petal. Scanning electron micrographs of cells from five of these regions, giving an overview of cellular diversity in this organ (scale bars=10μm). Region 8: conical cells; region 5: short trichomes; region 4: oblong cells; region 7: polygonal cells with smooth surfaces; region 3: secretory cells. Pictures are reproduced from Yao et al. (2019) with permission from the authors.

Other cell types are frequently found in the petal, and their distribution depends on their position in the petal. To explore this distribution along the petal proximo-distal axis, we chose the example of the petunia petal (*Petunia x hybrida*, Figure 1A). Petunia petals are fused, like petals from the vast majority of asterid flowers (Endress, 2001), and are organized in a tube and limbs (Figure 1A). In the limbs, cells are conical and smooth, and their density increases toward the center of the flower, which might influence petal color intensity and levels of emission of volatiles (Skaliter et al., 2021). At the most distal part of the tube, cells appear elongated and covered with striations (Figure 1A, tube 1). Striations are regular folds of the waxy cuticle of the outer epidermal cell wall and are frequently observed on petal epidermal cells (Antoniou Kourounioti et al., 2013). When regularly spaced and parallel oriented, these striations can cause light diffraction and iridescence of the petal, a visible cue for pollinators (Whitney et al., 2009b). Around the middle of the petunia petal tube, epidermal cells appear elongated with a small central papilla and still slightly striated (Figure 1A, tube 2). These striations progressively disappear as we progress toward the proximal part of the tube, and the central papilla becomes more and more pronounced (Figure 1A, tube 3). The function of this central papilla on tube cells is unknown.

Cell identity usually appears quite different on the two sides of the petal: Abaxial cells are flatter (lenticular) than adaxial conical cells, but they often contain pigments, and they can be a site of scent production (Kay et al., 1981; Baudino et al., 2007). Additionally, petal epidermal cells are often interspersed with trichomes, either glandular (for instance producing scent, nectar or defense compounds) or non-glandular ones, with various structures, shapes, and sizes. For instance in cotton flowers, both sides of the petals are covered in long non-glandular trichomes entangled together, resulting in the anchoring of adjacent petals together and their correct unfolding (Tan et al., 2016). In snapdragon flowers (*Antirrhinum majus*), glandular trichomes form very locally inside the corolla tube where they produce scent to attract pollinators and trap the pollen that they carry (Kolosova et al., 2001; Perez-Rodriguez et al., 2005). Finally, stomata are sometimes found on the petal epidermis, although their density is much more reduced than in leaves (Roddy et al., 2016; Zhang et al., 2018). They participate in gas exchange for photosynthesis in the petal (Zhang et al., 2018), and they might also be involved in maintenance of correct turgor pressure of the petal to avoid precocious wilting and have been proposed to play a role in flower opening in tulip (Azad et al., 2007).

This description of petal epidermal cell types is not exhaustive, and cell types in this tissue can be manifold. In elaborate petals, this diversity can be quite extreme. As an example, the *Nigella arvensis* flower forms highly elaborate petals of a complex shape with bifurcations and lobes, eyebrow-like stripes, long hairs, short trichomes, nectaries, and pseudo-nectaries (Figure 1B; Yao et al., 2019). Ten different subdomains can be defined in these petals, each displaying a distinct epidermal cell identity, among which conical cells, pavement cells, secretory cells, or polygonal cells, to cite just a few (Yao et al., 2019). One might argue that these petals are extremely derived and thus a particular case, but there is also strong variation in epidermal cell types on the petals of legume flowers, which are simple petals with a classical appearance (Dong et al., 2005; Ojeda et al., 2009).

## The Petal Mesophyll: Life and Death of the Petal

In between the two epidermises stands the petal mesophyll, a spongy tissue whose thickness greatly varies between species: a single-cell layer in poppies (van der Kooi and Stavenga, 2019) but several dozens in the giant *Rafflesia* flower (Nikolov et al., 2013; Mursidawati et al., 2020). The petal mesophyll comprises the vascular bundles of the petal, surrounded by parenchyma cells that are roundish cells without any striking visual features.

One obvious role of the mesophyll is for petal nutrition. Vascular bundles embedded within the parenchyma supply the water and metabolites necessary for petal function. Additionally, in some species like petunia, mesophyll parenchyma cells contain chloroplasts, even in the mature petal (Weiss et al., 1988; Vainstein and Sharon, 1993). Coupled to the presence of stomata on the petal epidermis and lacunae in the mesophyll favoring gas exchange, conditions are gathered for active photosynthesis to take place in petunia petals, although it is not as intense nor as efficient as in leaves (Weiss et al., 1988, 1990). This photosynthetic activity does not provide enough energy for the organ to be self-sustainable but, in particular, anthocyanin production appears to strongly depend on it (Weiss and Halevy, 1991).

The mesophyll is also involved in petal growth: In tulips, the mesophyll is considered to be the main driver of late petal growth by cell expansion (van Doorn and Van Meeteren, 2003), and in petunia, we recently showed that the mesophyll is the main driver for the growth of the petal tube (mainly by cell expansion), similarly to what had been previously observed in snapdragon flowers (Perbal et al., 1996; Efremova et al., 2001; Vincent et al., 2003; Chopy et al., 2021). In tulips and crocus flowers, temperature variation between lighted (outer) and shaded (inner) parts of the petal causes differential expansion of the parenchyma cell layers, resulting in flower opening (Wood, 1953). Similarly, in rose flowers, endoreduplication of parenchyma cells specifically on the adaxial side of the petal base, under the control of ethylene signaling, results in asymmetric growth of the petal mesophyll and flower opening (Cheng et al., 2021). Interestingly, only parenchyma cells toward the adaxial side of the petal respond to ethylene (Cheng et al., 2021), suggesting prior differentiation of mesophyll cells along the adaxial-abaxial axis.

The mesophyll also participates in petal pigmentation and therefore possibly in pollinator attraction. For instance in wallflowers petals (*Erysimum*), the epidermis is pigmented but the parenchyma cells also contain many chromoplasts and large pigmented cytoplasmic vesicles (Weston and Pyke, 1999). In the blue-flowered members of the *Boraginaceae* and *Liliaceae* families, the parenchyma cells contain anthocyanins and are the main contributor to petal pigmentation (Kay et al., 1981). The mesophyll can also influence the appearance of petals by reflecting or diffusing light. For example, buttercup petals (yellow-colored *Ranunculus*) have a reflective starch-containing parenchyma cell layer just underneath their epidermis, participating to the glossy and reflective petal surface (Parkin, 1928, 1931; Vignolini et al., 2012; van der Kooi et al., 2017). By a similar mechanism, the mesophyll of poppies and kingcup (*Caltha palustris*) petals contains large air cavities, creating a difference in refractive indices of the petal tissues and therefore strong light reflection and scattering, participating to the shiny appearance of the petals (Whatley, 1984; van der Kooi and Stavenga, 2019).

Finally, mesophyll cells are often the first site of petal senescence (van Doorn and Woltering, 2008). In petunia and lilies, this process begins in the petal parenchyma as early as 2days after pollination, as evidenced by signs of autophagy (granules formation, loss of membrane integrity or expression of programmed cell death markers; Shibuya et al., 2013; Mochizuki-Kawai et al., 2015). This suggests that resource relocation after pollination, from the petal to the ovary, first relies on mesophyll degradation. In Iris flowers, mesophyll cell death begins at the apical part of the petal and progresses toward the base (van Doorn et al., 2003), suggesting that the mesophyll is not entirely homogeneous in this respect and that the process is influenced by petal polarity.

In summary, mesophyll cells play various specific roles over the course of petal development. Although parenchyma cells display only subtle differentiation features and therefore might not be classified into different cell types within this tissue, there can be a zonation of their activity and function along the different petal axes.

## From Organ Identity to Cell Identity

As proposed in the ABCE model of floral organ identity, petal identity is specified in a region of the floral meristem by expression of B-class genes in a floral context, defined by A- and E-class genes, most of them being MADS-box genes (Schwarz-Sommer et al., 1990; Coen and Meyerowitz, 1991; Pelaz et al., 2000; Causier et al., 2010; Thomson and Wellmer, 2019). This is generally well conserved within all angiosperms (Soltis et al., 2007; Irish, 2009). The question then arises as to how expression of a small number of MADS-box genes results in the specification of the different petal cell types that we have described in the previous paragraphs. To our knowledge, in the petal mesophyll, nothing specific is known about the molecular players downstream of MADS-box genes that could define cell identity. In contrast, the acquisition of cell identity in the petal epidermis has been well characterized at the molecular level, specifically for conical cells and trichomes. Interestingly, and although these two cell types can appear quite different, it might be relatively simple to switch from one to the other.

Major molecular players in conical cell formation are MIXTA and MIXTA-like proteins, belonging to the large group of R2R3-MYB (MYB proteins with two repeats of the MYB DNA-binding domain) transcription factors (TFs). *MIXTA* was first identified in snapdragon petals; it is sufficient to drive both conical cell and trichome differentiation when overexpressed in tobacco leaves, but since its endogenous expression pattern is only late during petal development, it only directs conical cell differentiation *in vivo* (Glover et al., 1998; Martin et al., 2002). Indeed, another *MIXTA*-like gene, *AmMYBML1*, is expressed early in the ventral petal, and because of this early expression, it directs both conical cell and trichome differentiation (Perez-Rodriguez et al., 2005). This suggests that conical cell and trichome specification processes are closely related to the molecular level, and that shifts in the spatio-temporal pattern of *MIXTA*-like genes expression are sufficient to drive conical cell and/or trichome specification, and therefore the patterning of these cell types at the petal scale.

More generally, the identities of various plant epidermal cell types are determined by MBW protein complexes, composed of one MYB TF, one bHLH TF, and one WD40 repeat protein (Ramsay and Glover, 2005; Robinson and Roeder, 2015). In the different species (mainly *Arabidopsis*, snapdragon, maize, and petunia) and tissues (root, leaf, seed, and flower) where these complexes have been studied, they can trigger the formation of different cell types (trichomes, stomata, pavement cells, or root hairs), the production of pigmentation (in the whole plant, the seed coat or the petal), or of other epidermal features (seed mucilage). The WD40 protein appears to have a general scaffolding role, and there has been only one WD40 protein identified per species, while there are few bHLH proteins and many different MYB proteins, thereby resulting in a combination of specific MBW complexes (Ramsay and Glover, 2005). In petals, the specific role of these complexes has been elucidated in particular when exploring the petal pigmentation patterns in different petunia lines. Production of anthocyanins in petunia petals is controlled by MBW complexes composed of the WD40 protein ANTHOCYANIN11 (AN11), the bHLH protein AN1, and an R2R3-MYB protein that can be any among AN2, AN4, DEEP PURPLE (DPL), or PURPLE HAZE (PHZ), which will, respectively, result in pigmentation in the limbs (AN2), in the tube and anthers (AN4), in the petal veins (DPL), or during blushing of the petal under high light (PHZ; Quattrocchio et al., 1993, 1999; de Vetten et al., 1997; Spelt et al., 2000; Albert et al., 2011). These complexes regulate the expression of several structural genes in the anthocyanin pathway (Quattrocchio et al., 1993; Huits et al., 1994). The diversity and specificity of action of each MYB protein grant high modularity to the petal pigmentation system and the potential to evolve subtle changes in pigmentation patterns while avoiding to loose anthocyanin production entirely (Ramsay and Glover, 2005). Cell identity and pigmentation are thus specified by similar protein complexes in the petal epidermis.

**Figure 2:**
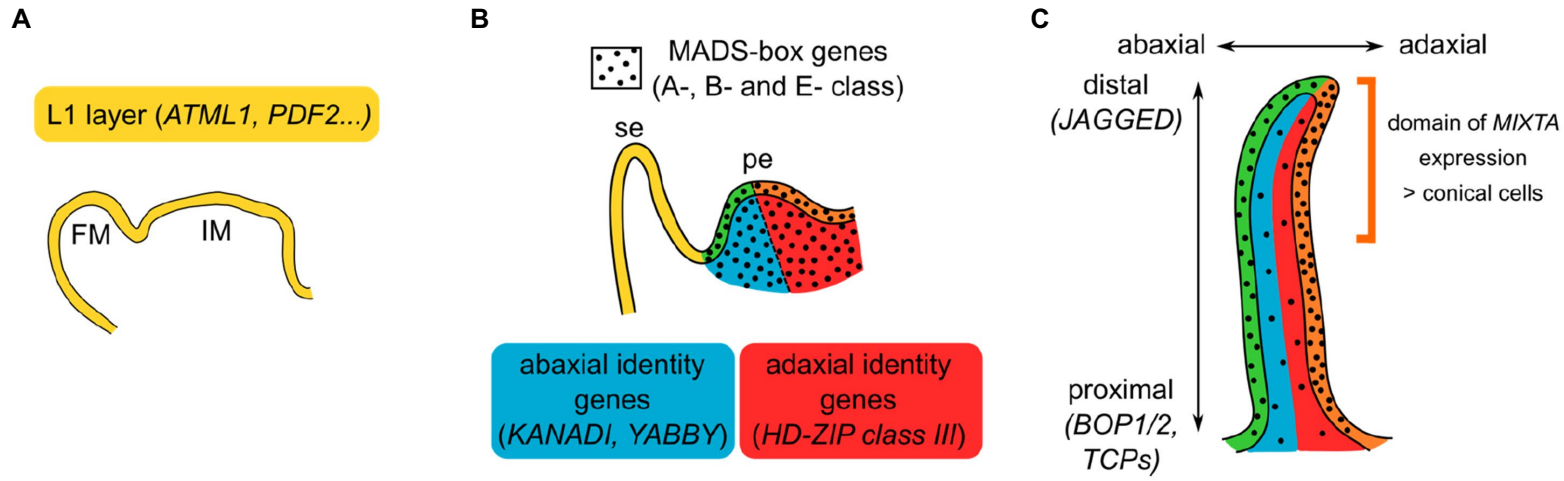
Model for the combinatorial specification of petal identity and polarities. **(A)** In the inflorescence and flower meristems (IM and FM, respectively), layer identity is already specified. In particular, the meristematic L1 layer and the epidermis that derives from it express a specific set of genes. **(B)** Initiating sepal (se) and petal (pe). Petal identity is defined by the expression of B-class MADS-box genes, in a floral context defined by A- and E-class genes, in all layers of the petal primordia (black dots). Abaxial (blue) and adaxial (red) sides of the petal are specified by a set of genes. Intersection of epidermal identity with abaxial or adaxial identity results in petal subdomains represented in green and orange, respectively. **(C)** Developing petal. Layer identity and abaxial/adaxial polarity are still maintained, and proximo-distal polarity establishes as the petal grows. MADS-box genes expression is not necessarily homogeneous in the developing petal: here is depicted the case of *Arabidopsis* SEP3 protein, enriched in the petal epidermis and particularly on its adaxial side. The combination of these different positional signals can result in the specification of distinct cell fates: In snapdragon, *MIXTA* is only expressed in the epidermal, adaxial, and distal part of the petal, driving there the formation of conical cells.

Additionally, the petal appears to be pre-patterned to specify particular cell fates when the right regulators are expressed at the right time and place (Figure 2). For instance, as seen previously, *MIXTA-like* genes do not direct the development of the same cell fates when expressed at a different time and place. What could this petal pre-patterning be? Briefly, markers of layer identity, such as the *HD-ZIP class IV* genes *MERISTEM L1 LAYER* (*ATML1*) or *PROTODERMAL FACTOR2* (*PDF2*) in *Arabidopsis* (Lu et al., 1996; Abe et al., 2003), specify epidermal identity from the embryonic stage onwards. Later, as floral organs initiate, their adaxial/abaxial polarity is established by genes, such as the *KANADI* and *YABBY* genes (abaxial side) and *HD-ZIP class III* genes (adaxial side; Siegfried et al., 1999; Kerstetter et al., 2001; Emery et al., 2003; Manuela and Xu, 2020), and their proximo-distal polarity is established by genes, such as *BLADE ON PETIOLE1* (*BOP1*) and *BOP2*, *TCP* genes or *JAGGED* (Hepworth et al., 2005; Norberg et al., 2005; Sauret-Gueto et al., 2013; Huang and Irish, 2015). More or less simultaneously, the B-class MADS-box genes specify petal identity, in a floral context specified by A- and E-class genes. Their initial expression appears quite homogeneous in all layers of the petal primordia (Urbanus et al., 2009; Prunet et al., 2017), but these genes are expressed throughout organ development and their expression pattern can be quite dynamic (Dornelas et al., 2011; Wuest et al., 2012). For instance in *Arabidopsis*, the E-class SEP3 protein is mostly expressed in the epidermis of the developing petal and more strongly on its adaxial side; similarly, the A-class AP1 protein accumulates more at the tip of developing sepals than at their base (Urbanus et al., 2009; Dornelas et al., 2011). Interestingly, mutations in epidermal specifier genes from the *PDF2* family result in alterations of petal identity with reduced expression of the B-class gene *APETALA3* (*AP3*), suggesting that *AP3* might be a particularly prominent target of these epidermal specifiers (Kamata et al., 2013a,b). MADS-box gene expression and/or protein localization might thus depend on layer identity, abaxial/adaxial, and proximal/distal polarity specifiers, through molecular mechanisms unknown so far. *Vice versa*, members from the *HD-ZIP class IV*, *KANADI, YABBY, HD-ZIP class III,* or *TCP* gene families, as well as *BOP1*, are found within the direct regulatory targets of B-class proteins in *Arabidopsis* (Wuest et al., 2012), suggesting a feedback loop between petal identity and positional signals within the petal.

How could these different positional signals relate to the different cell identities observed in the petal? Quite similarly to the combinatorial ABCE model proposed for floral organ identity, we propose that the combination of positional signals in the petal specifies the patterning of different cell types at the petal scale (Figure 2). The example of *MIXTA-like* genes, the main specifiers of conical cell fate, can illustrate this idea: In snapdragon, *MIXTA* is specifically expressed in the adaxial epidermis of the petal, particularly at the distal part where conical cells develop (Glover et al., 1998). This specific expression pattern can be interpreted as the result of the presence of petal and epidermal markers, together with distal and adaxial polarity specifiers. Indeed, pieces of genetic or molecular evidence support a link between *MIXTA-like* genes expression or function and positional signals: *MIXTA-like* gene expression is genetically downstream of petal identity, proximo/distal, and adaxial/abaxial specifiers (Eshed et al., 2001; Perez-Rodriguez et al., 2005; van Es et al., 2018), and MIXTA-like proteins can directly interact with HD-Zip class IV and TCP proteins (Yan et al., 2018; Camoirano et al., 2021). Therefore, one can imagine that petal positional signals activate *MIXTA-like* genes expression in the right time and place, driving cell differentiation toward the conical cell fate, later reinforced by the direct interaction of MIXTA-like proteins with proteins specifying position in the petal.

Downstream this layer of regulatory genes, effector genes act to modify the cytoskeleton arrangement and the cell wall, to give the petal cells their final shape and function, participating to their identity. Most of the knowledge on this topic comes from *Arabidopsis* conical cells, in which it was found that a circumferential arrangement of cortical microtubules, controlled by proteins such as KATANIN1, SPIKE1, or ROPs, supports cellulose deposition and cone formation (Ren et al., 2016, 2017). Other players, such as RHAMNOSE BIOSYNTHESIS 1, control cell wall composition in conical cells and thus correct cell and petal shape (Saffer et al., 2017), while striations on the surface of petal epidermal cells depend on enzymes from the cutin synthesis pathway (Li-Beisson et al., 2009). The direct link between those various effector genes and the upstream regulatory genes is not established yet, but a glimpse of the whole regulatory network is beginning to emerge (Irish, 2008; Huang and Irish, 2016). Additional molecular evidence is needed to understand how cell types are specified in the petal and surely, the processes of interest here are complex, continuous, and overlapping with each other, with extensive cross-talk involved throughout petal development.

## Conclusion and Future Directions

Although the petal is a simple laminar structure, it contains several different cell types whose identity is specified by a wide range of signals. How these signals are integrated at the molecular level and result in a specific gene expression profile and cellular function is mostly unknown. Today, the petal should not be viewed as an organ with a single identity, but rather as a population of cells in a petal specification context, each with a slightly different combination of lineage and positional signals (Xu et al., 2021). Single-cell technologies (transcriptome, proteome, interactome, chromatin accessibility, metabolome…) will surely lead to breakthroughs in the understanding of cell type specification in the petal and the molecular basis for its variation between species.

## Author Contributions

PM performed the electron micrographs. QC-S and MM wrote the article. All authors contributed to the article and approved the submitted version.

## Funding

This work is supported by grants to QC-S and MM from the Agence Nationale de la Recherche (grant ANR-19-CE13-0019, FLOWER LAYER).

## Conflict of Interest

The authors declare that the research was conducted in the absence of any commercial or financial relationships that could be construed as a potential conflict of interest.

## Publisher’s Note

All claims expressed in this article are solely those of the authors and do not necessarily represent those of their affiliated organizations, or those of the publisher, the editors and the reviewers. Any product that may be evaluated in this article, or claim that may be made by its manufacturer, is not guaranteed or endorsed by the publisher.
